# Psoriasis care in Germany: do patients who receive better care travel longer?

**DOI:** 10.1007/s43999-022-00008-0

**Published:** 2022-08-30

**Authors:** Nicole Mohr, Anna Langenbruch, Jobst Augustin, Natalia Kirsten, Matthias Augustin, Valerie Andrees

**Affiliations:** Institute for Health Services Research in Dermatology and Nursing (IVDP), University Medical Center Hamburg-Eppendorf (UKE), Martinistr. 52, 20246 Hamburg, Germany

**Keywords:** Psoriasis, Health services research, Choice of physician, Patient mobility, Travel time

## Abstract

**Background:**

Large variations in the quality of psoriasis care lead to patients being willing to bypass the nearest physician to receive higher quality of care. However, it remains unknown whether actual travel time is associated with quality of care. This study aimed to identify perceived quality of care determinants for travel time to the physician among patients with psoriasis in Germany. Furthermore, differences in access and perceived quality of care between urban and rural areas in Germany were analyzed.

**Methods:**

This cross-sectional observational study based on patient-level healthcare data. Perceived quality of care and treatment satisfaction were assessed from the patients’ perspective. Travel time was estimated by the patients. Multiple regression analysis with the predictors patient characteristics, system-related variables, urbanity, and patient satisfaction with treatment, was applied to identify determinants of travel time with subgroup analyses for rural and urban areas.

**Results:**

We included 497 patients from 29 dermatological practices in Germany. There were significant differences in psoriasis care between urban and rural areas. Longer travel time was associated with lower age, higher income, higher number of consulted dermatologists since diagnosis, rural residence, more waiting time for the first appointment, lower dermatologist density, and higher patient reported treatment satisfaction.

**Discussion:**

The results indicate an association between actual travel time and treatment satisfaction. Patients with higher perceived quality of care travel longer for their dermatological treatment. The results are also relevant to needs related planning.

## Introduction

Both German and international studies have demonstrated that there are large variations in psoriasis care [1, 2]. Internationally, such variations manifest for example in the marked differences in the use of different systemic therapies [1]. Systemic therapies are all forms of non-topic therapies, nonbiologic therapy, and biologic therapy [3]. If such variations cannot be explained by differences in illness, medical evidence, or patient preferences, they are defined as unwarranted variations [4]. The existence of unwarranted variations indicates potential inefficiencies and deficits and is reinforced by numerous potential barriers of care, which may vary locally. These include external barriers such as costs, workplace or accessibility of the physician but also the physician’s beliefs and safety concerns might play a role [5, 6]. Such barriers can be country-specific, for example due to the respective health system, but they also occur frequently on a smaller regional level: Small-area analyses have found within-country differences in quality of care. In Germany, for instance, the geographical accessibility of physicians can differ significantly between rural and urban areas [7]. In addition, regional differences in systemic treatment were reported as well as variations in outcome parameters such as disease severity, quality of life, and the patient-reported treatment benefit [2]. This indicates that deficits in care directly influence patient-reported outcomes such as quality of life or satisfaction with care.

As a chronic disease with various emotional and social needs, psoriasis requires long-term management with recurring physician consultations over an extended period of time [8, 9]. This causes many patients with psoriasis to be dissatisfied and frustrated with their disease management [10, 11]. In health systems with a free choice of provider, patients who are dissatisfied with their treatment tend to express their dissatisfaction by changing the physician [12]. Doing this, they often would accept long distances to the physician, which may be expressed as willingness-to-go [13]. Thus, determinants of travelled distance can be interpreted as indicators for what a patient values for his or her treatment. In case of psoriasis, patients stated in a survey that, hypothetically, they would be willing to bypass the nearest physician if they received higher quality of care and that they would accept longer distances than necessary [14, 15]. What remains unknown, however, is whether actual travel time is associated with quality of care.

Against this background the objective of the current analysis was to identify determinants of travel time to the physician among patients with psoriasis. A particular focus was on the influence of the perceived quality of care and treatment satisfaction. Furthermore, differences in accessibility and perceived quality of care and treatment satisfaction between urban and rural areas in Germany were analyzed.

## Materials and methods

### Study population

A cross-sectional study was conducted to gain detailed and representative information on routine care in adult patients with psoriasis. Data were collected via dermatologists in Germany and included reports from the dermatologists themselves as well as self-reports from patients. The study site selection based on the total list of dermatologists in Germany as used in previous healthcare studies [16, 17]. A random sample of *N* = 73 dermatologists was invited to participate. Of those, *n* = 29 dermatologists submitted completed questionnaires. The dermatologists were asked to include sequentially every patient fulfilling the inclusion criteria (age ≥ 18 years, clinically diagnosed psoriasis vulgaris, written informed consent) regardless of severity or therapy.

This study is part of the “PsoBarrier EU” project (ClinicalTrials.gov NCT02668341), which examines barriers of guideline-compliant treatment of psoriasis on different levels in five European countries.

### Data collection and covariates

Data were collected between January 2016 and December 2017 using two standardized questionnaires, one to be completed by the patient and one by the dermatologist, respectively.

#### Individual patient characteristics

Sociodemographic variables obtained with the patient questionnaire were age (in years), gender (male or female), highest educational degree, employment status, household income and the number of persons living in the household. The highest educational degree was dichotomized for further analyses. We derived the net equivalence income from the household income divided by the number of persons in the household. For this calculation the first adult person had a weight of 1, each further adult 0.5 and children 0.3 (OECD-modified equivalence scale) [18].

Additionally, the number of dermatologist consultations within the last 12 months, the number of different consulted dermatologists since the diagnosis, inpatient treatment in the last five years and workdays lost due to psoriasis were obtained with the patient questionnaire. Further patient-reported outcomes were quality of life (QoL) determined by the Dermatology Life Quality Index (DLQI; range 0–30; 0 = no quality of life impairment; 30 = maximum impairment of QoL) [19] and the self-reported health state determined by the EuroQol visual analogue scale (EQ VAS; range 0–100; 0 = worst imaginable health; 100 = perfect health) [20]. Information on previous treatments within the last five years was provided by the patient, current therapy by both the patient and the physician.

Severity of psoriasis was determined by the dermatologist using the Psoriasis Area and Severity Index (PASI; range 0–72; 0 = no severity; 72 = maximum disease) and body surface area (BSA; percentage of body surface with skin lesions). According to the German S3 treatment guideline, moderate to severe psoriasis was defined as PASI > 10 or BSA > 10 and DLQI > 10 [21].

#### Geographic data

The locations of both the patients and the dermatologists were available on ZIP code level. The ZIP codes were assigned to the corresponding counties, which in turn were categorized into combined county types. This categorization was carried out according to the settlement structure types of the Federal Institute for Research on Building, Urban Affairs and Spatial Development (Bundesinstitut für Bau-, Stadt- und Raumforschung, BBSR) into four groups: 1) counties being big cities, 2) urban counties, 3) rural counties showing densification and 4) sparsely populated rural counties [22]. These county types were dichotomized for further analyses: Counties being big cities and urban counties were summarized to urban areas, while rural counties showing densification and sparsely populated rural counties were summarized to rural areas.

The density of dermatologists on county level was derived from the Central Research Institute of Ambulatory Health Care in Germany [23]. Travel time to the dermatologist, travel costs and waiting time for the first appointment with the current dermatologist were estimated by the patients. In addition to the actual travel time, hypothetical waiting and travel time that patients consider acceptable were determined. The latter is referred to as maximum willingness-to-go in this paper.

#### Perceived quality of care and treatment satisfaction

The perceived quality of care and treatment satisfaction were assessed directly from the patients and by analyzing the mean online ratings gathered from two physician rating websites.

Indicators from the patients’ perspective were the categorial variables “How satisfied have you been with the treatment of your psoriasis over the past 12 months?” (Scale from 1 = very dissatisfied to 4 = very satisfied) and “How well did you feel informed about psoriasis by the physician?” (Scale from 1 = very poorly informed to 5 = very well informed). In order to obtain information on the extent to which patients are involved in treatment and therapy decisions, the questionnaire asked how well patients felt informed about psoriasis (ranging from 1 = very poorly informed to 5 = very well informed) and whether they defined therapeutic goals together with their dermatologist (yes, no/unsure) as suggested in the German S3 treatment guideline and the European consensus on psoriasis [21, 24].

Additionally, the mean online ratings gathered from two commonly used physician rating websites (jameda and sanego) were analyzed for 2019. On these websites, patients can rate their experience at the practice in several questions on treatment satisfaction, trust relationship with the physician, time taken by the physician, friendliness of staff, and medical information. From these questions, the websites derive one mean value for each patient’s evaluation. We then calculated a score for this analysis by weighting the average online ratings by the total number of ratings of each website. This score ranged from 0–10, with higher values indicating better online rating. Each of the 29 centers in our study has one rating.

### Statistics

Data were described using standard statistical parameters (relative frequencies for categorical data; mean and standard deviation for continuous data). Differences between subgroups were analyzed using the χ^2^ test for dichotomous data and the t-test for continuous data. Missing data were not replaced by any values. Statistical significance was set at a *p* value of < 0.05.

A linear regression analysis was conducted to identify parameters that were associated with longer travel time. We chose included variables based on a previous conducted scoping review on willingness-to go and physician choice. Here, a conceptual framework was developed that identified different variables concerning access, quality of care, and the individual patient level as predictors for the willingness to travel further distances for medical treatment. We included those predictor variables on access, quality of care and the individual patient, that were available for the analysis [13]. The estimated travel time was the dependent variable. Cases with travel time longer than 120 min were excluded in accordance with comparable studies [25]. School certificate, waiting time for the first appointment > 1 month, setting therapeutic goals together with the dermatologist, were dichotomized variables. For waiting time and setting goals together, the answer “don’t know” was counted as “no” for this analysis to avoid a high number of missing values. Additionally, we determined the mean patient defined treatment satisfaction per dermatological center and correlated this with the online rating per practice.

All analyses were conducted using SPSS Version 27.0 (IBM Corp., Armonk, NY, USA).

## Results

### Study population

In total, 29 dermatological practices in Germany participated and included *N* = 502 patients, resulting in *N* = 497 complete data sets (both patient and physician questionnaire completed). All but one regional Association of Statutory Health Insurance Physicians in Germany were covered. Mean age of the patients was 49.7 ± 14.9 years and 41.4% were female. For 19 (3.8%) patients, no ZIP code or an invalid ZIP code was available, thus these patients were excluded from the regional analyses. From the remaining 478 patients, *n* = 390 (81.6%) lived in urban and *n* = 88 (18.4%) in rural areas. These two subgroups were comparable regarding sociodemographic characteristics. Following the definition of the German S3 treatment guideline, 12.1% had moderate to severe psoriasis.

**Fig. 1:**
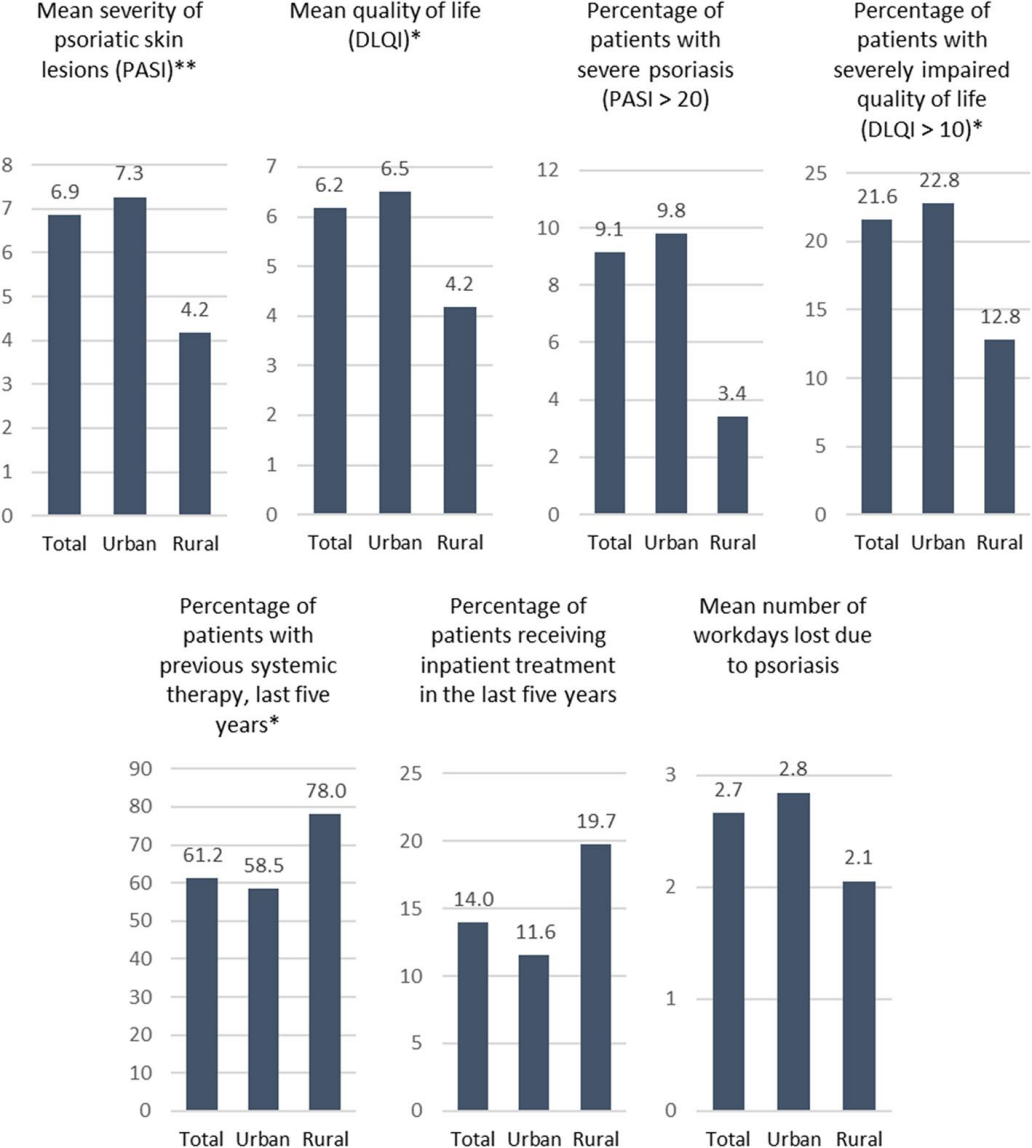
Health care quality indicators in total and compared between urban and rural areas (differences according to χ^2^ or t-test: * *p* < 0.05; ** *p* < 0.001), different y-axis scales need to be noted

Patients living in rural areas were diagnosed with significantly lower disease severity according to the mean PASI and had less impaired quality of life (mean DLQI and a lower percentage of patients with a DLQI > 10, Table 1). Furthermore, the proportion of patients with previous systemic therapy and inpatient treatment in the last five years were significantly higher in rural areas. Whereas the mean number of lost working days due to psoriasis was higher in urban areas (Fig. 1).

**Table 1:** Characteristics of the psoriasis study population and subgroups by county type of the patient residence

	**Total sample (*N* = 497)**	**Combined county type**	
		**Urban areas (*n* = 390)**	**Rural areas (*n* = 88)**
Female (%)	41.4	43.3*	31.8*
Mean age ± SD (years)	49.7 ± 14.9	48.6 ± 15.0**	53.9 ± 13.5**
Abitur (German secondary school certificate qualifying for university admission, % yes)	27.8	30.2	19.8
Mean working hours per week ± SD	23.8 ± 19.8	23.7 ± 19.6	23.9 ± 20.8
Mean net equivalence income ± SD (€)	1,676.2 ± 933.5	1,660.2 ± 939.3	1,728.7 ± 874.6
Mean number of dermatologist consultations in the last 12 months ± SD	7.1 ± 14.5	7.2 ± 16.1	6.8 ± 7.5
Mean number of different dermatologists consulted since first diagnosis of psoriasis ± SD	3.0 ± 2.3	3.0 ± 2.4	3.1 ± 2.0
Mean self-assessed health state (EQ VAS) ± SD	69.0 ± 21.4	68.8 ± 21.7	70.4 ± 20.1
Psoriasis Area and Severity Index (PASI)			
Mean ± SD	6.9 ± 8.4	7.3 ± 8.6***	4.2 ± 5.9***
PASI > 10 (%)	22.4	24.4**	10.3**
Dermatology Life Quality Index (DLQI)			
Mean ± SD	6.2 ± 6.8	6.5 ± 6.8**	4.2 ± 6.2**
DLQI > 10 (%)	21.6	22.8*	12.8*
Waiting time for first appointment, more than 1 month, (“don´t know” excluded, %)	16.5	15.7	19.4
Mean dermatologist density, n per 100.000 ± SD	4.2 ± 1.5	4.6 ± 1.5***	2.9 ± 1.0***
Feeling informed about psoriasis ± SD	4.0 ± 0.9	4.0 ± 0.9	4.1 ± 1.0
Defining therapeutic goals together with the dermatologist, yes (%)	32.0	29.5**	43.9**
Satisfaction with treatment of the past 12 months ± SD	3.2 ± 0.8	3.1 ± 0.8**	3.4 ± 0.7**

*SD* Standard deviation, *EQ VAS* EuroQol visual analogue scale, *DLQI* Dermatology Life Quality Index, *PASI* Psoriasis Area and Severity Index

^*^ *p* < 0.05; ** *p* < 0.01 *** *p* < 0.001 (difference between urban and rural areas)

Missing values in total sample due to unavailable ZIP code data in combined county types

### Perceived quality of care and patient satisfaction

Overall, 75.4% felt well or very well informed about their psoriasis. Regarding patient involvement, 32.0% stated that they define therapeutic goals with their physician, 68% did not or did not know. To define therapeutic goals with the dermatologist was stated more often by patients from rural countries (Table 1).

**Fig. 2:**
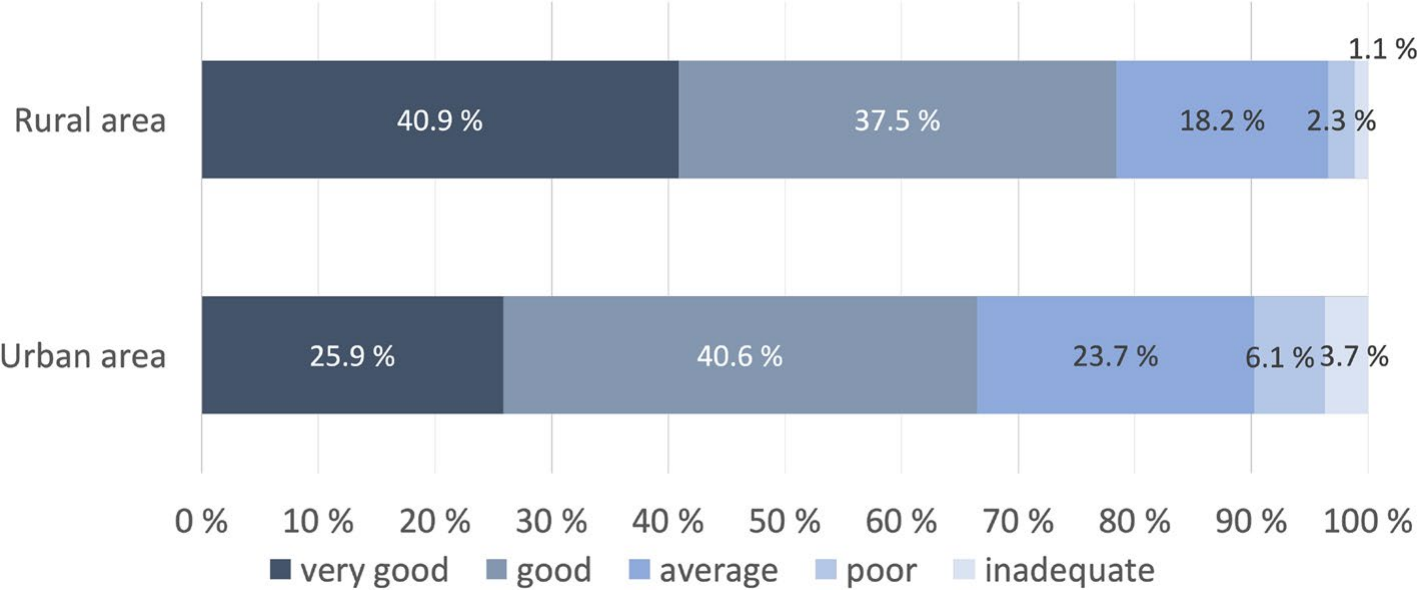
Rating of health services over the past years of patients living in rural (*n* = 88) and urban areas (*n* = 379)

**Fig. 3:**
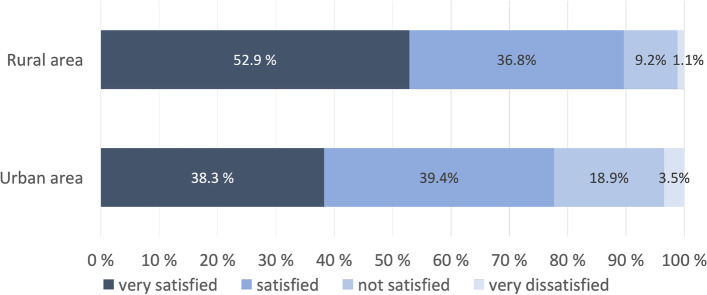
Satisfaction with the treatment over the past 12 months of patients living in rural (*n* = 87) and urban areas (*n* = 376)

Of the total sample, 68.5% rated the health services provided over the last years as very good or good (Fig. 2). This proportion differed significantly (*p* = 0.030; χ^2^ test) between patients living in rural (78.4%) and urban areas (66.5%). Satisfaction with the treatment of psoriasis over the past 12 months was also higher in rural areas than in urban areas (Table 1 and Fig. 3).

### Physician online ratings

The mean online rating of the 29 participating dermatologists was 6.9 ± 2.4 (scale from 0 [worst possible rating] to 10 [best possible rating]; minimum rating 0.4; maximum rating 9.8). The online rating did not correlate with the mean patient-reported satisfaction with treatment over the past 12 months per center (*r* = 0.200; *p* = 0.326, *n* = 26 dermatological centers) and did not differ between rural and urban locations of the centers (6.7 ± 2.5 vs. 7.6 ± 1.3, *p* = 0.500, *n* = 26 dermatologists).

### Waiting time

The majority of the patients (58.8%) had a waiting time for the first appointment with their current dermatologist of less than two weeks. One month or longer was indicated by 16.5%. Concerning the waiting time, there was no significant difference between urban and rural areas (Table 1).

Patients stated that they would be willing to wait a maximum of 3.3 ± 2.3 weeks for an appointment with a psoriasis specialist. In terms of the hypothetical waiting time, patients in rural areas specified a longer waiting time as acceptable than patients in urban areas (3.9 ± 3.2 vs. 3.2 ± 2.1 weeks; *p* = 0.017). Furthermore, there was a small negative correlation between the acceptable waiting time and the PASI (*r* = -0.125; *p* = 0.011), indicating that patients with less severe disease accept a longer waiting time for the dermatologist.

### Travel time

The mean travel time and travelled distance to the dermatologist was 27.2 ± 22.9 min and 18.2 ± 21.8 km, respectively. One third of the patients (32.6%) visited a dermatologist in a different county than their place of residence. The estimated travel costs were 7.0 ± 8.9 euros. Travel time, travelled distance and travel costs were significantly higher in rural areas compared to urban areas. Regarding hypothetical travel time, patients in rural areas had a significantly higher willingness-to-go than patients in urban areas (Table 2).

**Table 2:** Realized and hypothetical travel time, stratified by combined county type

	**Total sample (*N* = 497)**	**Combined county type**	
		**Urban areas (*n* = 390)**	**Rural areas (*n* = 88)**
Mean travel time ± SD (min)	27.2 ± 22.9	24.1 ± 20.0***	41.4 ± 30.2***
Mean travelled distance ± SD (km)	18.2 ± 21.8	14.1 ± 15.4***	33.8 ± 33.7***
Mean estimated travel costs ± SD (€)	7.0 ± 8.9	5.6 ± 6.6***	12.7 ± 13.8***
Mean maximum willingness-to-go ± SD (min)	58.1 ± 46.8	54.8 ± 48.6***	71.8 ± 39.2***

*SD* Standard deviation

^***^ *p* < 0.001

Missing values in total sample due to unavailable ZIP code data in combined county types

Linear regression analysis (adjusted *R*^*2*^ = 0.224; *n* = 276) revealed that higher age and higher dermatologist density were associated with shorter travel time. A higher net equivalence income, a higher number of different consulted dermatologists since the first diagnosis, a longer waiting time for the first appointment, rural residence, and higher treatment satisfaction were significantly associated with longer travel time (Table 3).

**Table 3:** Linear regression model, dependent variable: travel time in minutes, *n* = 276, adjusted *R*^*2*^ = 0.224

**Domain**	**Determinants**	**B**	**95%CI**		**Beta**	***p***
			**Lower**	**Upper**		
Individual patient	Sex, female	2.38	-2.66	7.43	0.06	0.353
	**Age, in years**	-0.26	-0.43	-0.09	-0.19	0.003
	Abitur (certificate from German secondary school qualifying for university admission), yes	-1.44	-6.74	3.86	-0.03	0.593
	Working hours per week, in hours	0.00	-0.14	0–14	0.00	0.969
	**Net equivalence income, in €**	0.00	0.00	0.01	0.16	0.021
	Number of dermatologist consultations, last 12 months, n	0.02	-0.16	0.20	0.01	0.843
	**Number of different dermatologists consulted since first diagnosis of psoriasis, n**	1.13	0.15	2.12	0.13	0.024
	Self-assessed health state (EQ VAS), range 0–100^a^	-0.12	-0.25	0.02	-0.12	0.082
	PASI, range 0–72^b^	-0.11	-0.45	0.23	-0.04	0.537
	DLQI, range 0–30^c^	0.23	-0.27	0.74	0.07	0.363
Access	**Residence, rural region**	12.12	5.55	18.69	0.23	< 0.001
	**Waiting time for first appointment, more than 1 month**	7.49	0.14	14.83	0.11	0.046
	**Dermatologist density, n per 100.000**	-2.97	-4.51	-1.43	-0.24	< 0.001
Quality of care from patient’s perspective	Feeling informed about psoriasis, range 1–5^d^	0.63	-2.18	3.44	0.03	0.659
	Defining therapeutic goals together with the dermatologist, yes	3.62	-1.51	8.76	0.08	0.166
	**Satisfaction with treatment of the past 12 months, range**^e^	4.86	8.49	1.22	0.19	0.009

^a^EuroQol visual analogue scale (EQ VAS): scale from 0 = worst imaginable health to 100 = best imaginable health)

^b^Psoriasis Area and Severity Index (PASI): scale from 0 = no severity to 72 = maximum severity

^c^Quality of life (DLQI): scale from 0 = no quality of life impairment to 30 = maximum quality of life impairment

^d^Feeling informed about psoriasis: scale from 1 = very poorly informed to 5 = very well informed

^e^Satisfaction with treatment of the psoriasis over the past 12 months: scale from 1 = very dissatisfied to 4 = very satisfied

## Discussion

The rationale for the current study was to identify determinants of travel time to the dermatologist among patients with psoriasis in Germany. This objective is built on previous studies that indicated that patients with psoriasis are willing to take on more distance, time, and costs for good treatment [14, 15].

The data revealed that individual patient characteristics as well as treatment satisfaction and access variables are significantly associated with travel time to the dermatologist. Furthermore, we found that there are differences in psoriasis care between urban and rural areas in Germany.

Perceived quality of care was examined by direct patient assessment and supplemented by the use of web-based physician ratings which are publicly available. As in most cases, patients are no medical experts, a lack of information on quality can be assumed. Consequently, patients might not make optimal choices [26]. Therefore, our findings on quality of care from the patients’ perspective are mostly limited to the perceived quality and do not necessarily have to mirror objective quality. Nevertheless, perceived quality of care has constantly been shown to be the most important factor for choosing a physician [14, 15]. In a cross-sectional study conducted in 2013, 25% of the patients had already used physician rating websites for choosing a physician and popularity is expected to increase [27]. Although these ratings are most likely to be biased with an overrepresentation of negative evaluations and commonly doubted to reflect clinical quality of care, they have been shown to be associated with the satisfaction of the patients and to influence patients’ choice of physician [28–30]. The association between satisfaction with treatment and online-rating results per center was not identified in our data. In the regression analysis, treatment satisfaction as an indicator for the perceived quality of care was a predictor for travel time. This indicates that patients value to be involved in therapy decisions and, therefore, strengthens considerations on the importance of shared decision making.

All three included determinants from the domain access (degree of urbanity, waiting time, and dermatologist density) were significantly associated with travel time. These results were expected and plausible and have already been shown in other studies [25]. Furthermore, younger patients and those with higher net equivalence income had longer travel time.

Our results further indicate that quality of care is rated better by patients from rural areas than by patients from urban areas. This was observed from the individual patient defined treatment satisfaction and not by the online ratings for the centers from rural and urban areas. The perceived quality of care is likely to be biased by what patients expect from their treatment. In this regard, our results suggest that these expectations might be substantially different among patients living in rural or urban areas. For example, patients in rural areas considered much longer waiting and travel time as acceptable compared to patients in urban areas. Furthermore, expectations are substantially influenced by past experiences. This is also backed by our finding that patients who visited many different dermatologists, and therefore might have a longer history of bad experiences, travelled longer to see their current physician.

The percentage of patients receiving systemic treatment in the last five years was significantly higher in rural areas. Previous studies already suggested that prescription rates of systemic treatment are higher in regions that are less densely populated and found associations of regions with clinical severity and quality of life [2]. One could speculate whether this might be related to the higher effort associated with consulting a physician in rural areas. However, at least in our data, the frequency of dermatologist consultations did not differ between rural and urban areas. Further possible explanations for differences in the prescription rates might be budget restraints for physicians or their different levels of knowledge about the German S3 treatment guideline.

A limitation with regard to interpreting differences between rural and urban areas is the small number of patients in rural areas, which does not allow further stratification. Therefore, we could not control for economic and regulatory conditions such as those presented in other studies [2]. Another limitation was that the location of the patients and the dermatologists were only available on ZIP code level. Exact address data would be desirable here as it would allow to perform geographical network analyses and further spatial analyses. The study was designed to provide a representative sample of adult patients in psoriatic treatment in Germany and to illustrate routine care. For this, data collection was carried out in different practices all over Germany. Nevertheless, regarding the rather low response rate of physicians (about 40%), it can be assumed that participating physicians might be more engaged than the ones, who did not participate. They might not be representative, which needs to be considered when interpreting the results.

Overall, the results support previous findings suggesting that there is an association between quality of care and travel time [14]. Although this study is limited by not knowing if patients bypass the nearest physicians to receive better care, travel time is a strong indicator. We used data from Germany as an example, however, the results are assumed to be also valid for other countries with a free choice of the provider. Our results indicate that treatment satisfaction is crucial for the willingness-to-go, given that a patient is physically and financially able to make an increased effort. This leads to further discussions on whether access to high quality care is subject to equity considerations. In the UK for example, researchers showed a social gradient in those who decide to travel beyond the local area for treatment [31]. The data from our study provide important insights into patient mobility and thus form the basis for further research on willingness-to-go and bypassing. Online rating portals seem to have an impact on the choice of the physician in the first place but in our study, they did not correlate with treatment satisfaction. In this context it would be beneficial to examine, if these portals reflect the actual quality of care and, if necessary, how the quality of information provided by these portals can be improved. A further conclusion is that currently no homogeneous care for psoriasis is offered. It is up to the patients to overcome barriers to care [6] by increasing their willingness-to-go. Here, empowering patients to take on responsibility for their treatment is essential. For further research, the question of whether increasing mobility is worthwhile in terms of improving quality of care remains. In this context it would be interesting to investigate how disease severity and health related quality of life develop longitudinally as this cross-sectional design has limitations to explain causalities. The results are also relevant to need related planning, especially with regard to acceptable driving and waiting time.

## Data Availability

The datasets used and/or analyzed during the current study are available from the corresponding author on reasonable request.
